# Anthropometric indicators and dental caries in the pediatric population of rural communities in the peruvian amazon: a cross-sectional study

**DOI:** 10.21142/2523-2754-1304-2025-264

**Published:** 2025-11-08

**Authors:** Christian Renzo Aquino-Canchari, Ekaterina Fabiola Parraga Camayo, Estrellita Naomi Ambrosio Huaman, José Alejandro Román Santiago

**Affiliations:** 1 Universidad de Huánuco (UDH). Huánuco, Perú. Christian.aquino.canchari@gmail.com Universidad de Huánuco Universidad de Huánuco (UDH) Huánuco Peru Christian.aquino.canchari@gmail.com; 2 Universidad Continental, Facultad de Medicina Humana, Sociedad Científica Médico Estudiantil Continental (SOCIMEC). Huancayo, Perú. 72535695@continental.edu.pe 73027843@continental.edu.pe 71504647@continental.edu.pe Universidad Continental Facultad de Medicina Humana Sociedad Científica Médico Estudiantil Continental (SOCIMEC) Huancayo Perú 72535695@continental.edu.pe 73027843@continental.edu.pe 71504647@continental.edu.pe

**Keywords:** dental caries, body mass index, nutritional status, rural population, child dental care, childhood obesity, fat distribution, caries dental, índice de masa corporal, estado nutricional, población rural, atención dental para niños, obesidad infantil, distribución de grasa corporal

## Abstract

**Introduction:**

Dental caries and nutritional alterations are common in childhood, particularly in rural communities with limited access to health services.

**Objective:**

To determine the association between anthropometric indicators and the presence of dental caries in children aged 5 to 11 years from selected rural communities in the Peruvian Amazon.

**Methods:**

Cross-sectional study including 116 children from Llaylla, Pampa Hermosa, and Belén (Satipo, Junín). Body mass index for age (BMI/A), abdominal circumference for age (AC/A), and height for age (H/A) were assessed according to WHO and MINSA standards. Dental caries were evaluated using the DMFT/dmft index and the clinical consequences of untreated dental caries (pufa/PUFA). Associations between anthropometric variables and dental caries were analyzed using logistic regression adjusted for age and sex, with p<0.05 considered statistically significant.

**Results:**

Children with overweight/obesity had a higher risk of caries in permanent dentition (OR=3.875) and a lower risk in primary dentition (OR=0.539). Those with high or very high risk according to AC/A showed OR=0.968 in permanent dentition and OR=0.864 in primary dentition. Children with low height for age (H/A) presented OR=1.319 in permanent dentition and OR=1.757 in primary dentition. All associations were not statistically significant (p>0.05).

**Conclusion:**

Dental caries is highly prevalent among Amazonian children. Anthropometric indicators were not significantly associated with caries, suggesting the influence of other factors such as diet and access to dental care.

## RESUMEN

**Introducción:**

La caries dental y las alteraciones nutricionales son comunes en la infancia, particularmente en comunidades rurales con acceso limitado a servicios de salud.

**Objetivo:**

Determinar la asociación entre indicadores antropométricos y la presencia de caries dental en niños de 5 a 11 años de algunos centros poblados rurales de la selva peruana.

**Materiales y métodos:**

Estudio transversal en 116 niños de Llaylla, Pampa Hermosa y Belén (Satipo, Junín). Se evaluaron el peso, la talla y el perímetro abdominal para calcular IMC/E, PAB/E y T/E, según estándares de la OMS y el Minsa. La salud bucal se determinó mediante DMFT/dmft y pufa/PUFA. La asociación entre variables antropométricas y caries se analizó mediante regresión logística ajustada por edad y sexo, considerando p < 0,05 como significativo.

**Resultados:**

: Se evidenció que los niños con sobrepeso u obesidad presentaron un mayor riesgo de caries en dentición permanente (OR = 3,875) y un menor riesgo en dentición decidua (OR = 0,539). Aquellos con alto o muy alto riesgo, según el perímetro abdominal para la edad (PAB/E), mostraron OR = 0,968 en dentición permanente y OR = 0,864 en dentición decidua; mientras que los niños con baja talla para la edad (T/E) presentaron OR = 1,319 en dentición permanente y OR = 1,757 en dentición decidua. Ninguna de las asociaciones mostró significancia estadística (p > 0,05).

**Conclusión:**

La caries dental es altamente prevalente en niños amazónicos. Los indicadores antropométricos no se asociaron significativamente con caries, lo que sugiere la influencia de factores como la dieta y el acceso a servicios odontológicos.

## INTRODUCTION

Oral health is an essential component of overall well-being, and its assessment in children and adolescents provides a comprehensive understanding of their health status ^1^. In rural and Amazonian areas of Peru, as in other developing countries, oral health is influenced by a variety of socioeconomic and environmental factors ^2^.

In this context, anthropometric indicators such as weight, height, weight-for-age, height-for-age, and body mass index (BMI) constitute fundamental parameters for evaluating child growth and development. Their relationship with general health is well documented ^3^. However, the specific association between these indicators and oral health in children remains poorly explored, particularly in rural populations of the Peruvian Amazon.

The literature suggests that in regions with socioeconomic limitations and particular environmental conditions, the interaction between nutritional status and oral health may be highly relevant ^4^^,^^5^. Inadequate nutrition, reflected in alterations of anthropometric indicators, has been associated with an increased risk of dental diseases such as caries and periodontal infections ^6^^,^^7^. Additionally, factors inherent to the Amazonian environment, including the availability and type of foods consumed, affect both nutrition and oral health in children ^7^^,^^8^.

Therefore, the present study aims to evaluate the association between anthropometric indicators and dental caries in the pediatric population of rural communities in the Peruvian Amazon, contributing evidence on how these variables may interact and affect child health

## MATERIAL AND METHODS

The study was approved by the Institutional Ethics Committee of Universidad Continental (Official Letter No. 0556-2024-CIEI-UC). Informed consent was obtained from parents or legal guardians, and assent was provided by the children through an age-appropriate assent form. All data were handled confidentially, ensuring participant welfare at all times.

An observational cross-sectional study was conducted within the framework of the Multidisciplinary University Research and Service Camp (CUMIS), organized by SOCIMEC in 2024. The study population included 116 children aged 5 to 11 years, from the rural communities of Llaylla, Pampa Hermosa, and Belén, in the district of Llaylla, Satipo province, Junín. Participants attended the nutritional and dental care module established during the camp.

All children were included in the study after obtaining informed consent from parents or guardians and assent from the participants. Exclusion criteria applied only to cases where reliable data could not be obtained: for anthropometric evaluation, children with physical limitations, lack of cooperation, or use of devices that interfered with measurements; for dental evaluation, children with incomplete clinical records, lack of cooperation during intraoral examination, or presence of acute pain or lesions that prevented proper visual inspection.

### Variables

#### Anthropometric indicators

Body mass index-for-age (BMI/A): calculated by dividing weight in kilograms by height in meters squared, adjusted for the child’s age and sex ^13^^,^^14^. BMI/A was classified following WHO Z-score categories: underweight (< -2 SD), normal (-2 to +1 SD), overweight (> +1 to +2 SD), and obesity (> +2 SD) ^9^.

Abdominal circumference-for-age (AC/A): assessed using reference percentiles established by the Peruvian Ministry of Health (MINSA), adjusted for age and sex. This anthropometric indicator identifies the risk of abdominal obesity, a predictor of metabolic and cardiovascular complications. According to official classification, it was defined as no modified risk (RSM) when AC/A was below the 75th percentile (<P75), increased risk (RSA) when between the 75th and 89th percentiles (≥P75 and <P90), and very increased risk (RMA) when equal to or above the 90th percentile (≥P90). For example, in a 5-year-old girl, an AC/A ≥ 64,4 cm corresponds to RMA (≥P90), while values between ≥ 59,1 cm and < 64,4 cm are classified as RSA (≥P75 and <P90) ^10^.

Height-for-age (H/A): calculated from the relationship between measured height and chronological age, according to WHO Growth Standards 2006 and 2007 ^11^^,^^12^. Growth retardation was considered when the Z-score was < -2 SD. Classification was as follows: Z < -3 SD: severe stunting (severe growth retardation); -3 SD ≤ Z < -2 SD: stunting (growth retardation); -2 SD ≤ Z ≤ +2 SD: normal height-for-age; Z > +2 SD: tall stature-for-age ^9^.

#### Oral Health Assessment

Oral health was evaluated using two complementary indicators:

Caries experience: determined according to WHO criteria and classified as very low (0,1-1,1), low (1,2-2,6), moderate (2,7-4,4), high (4,5-6,5), and very high (>6,6). Incipient lesions (white spots) were not considered ^11^^,^^12^.

Clinical consequences of untreated dental caries: assessed using the pufa/PUFA index, which sums teeth with pulpal involvement (p/P), ulceration (u/U), fistula (f/F), and abscess (a/A). In cases of diagnostic uncertainty, the lowest score was assigned ^13^.

Data collection was performed using an epidemiological record designed according to WHO guidelines ^14^. Examinations were conducted under natural light, which may have limited the detection of incipient lesions; however, examiners were previously calibrated and trained to ensure reliability. Mirrors and explorers were used only as visual aids, and non-cavitated lesions were not recorded, ensuring comparability with previous studies.

### Procedures

Anthropometric evaluation included weight measurement using a calibrated scale (precision 0,1 kg) with participants barefoot and wearing light clothing; height, recorded with a stadiometer in an upright position and barefoot; and abdominal circumference, measured with a flexible measuring tape at the level of the navel at the end of a normal exhalation.

Dental examination was performed via direct clinical inspection on a chair with back support, under natural light, using basic instruments (mirror and explorer). Prior to the examination, teeth were cleaned with moistened gauze. The presence of caries, gingival lesions, periodontopathies, ulcers, fistulas, and abscesses was recorded.

### Statistical analysis

Data were recorded on clinical forms specifically designed for the study, then entered into Microsoft Excel 2019 using double entry to minimize errors, and subsequently analyzed in Stata version 16. Measures of central tendency and dispersion were calculated for continuous variables, while absolute and relative frequencies were determined for categorical variables. The association between anthropometric indicators and dental caries was assessed using binary logistic regression, entering all anthropometric variables (body mass index, height-for-age, and abdominal circumference) simultaneously using the enter method. Odds ratios (OR) with 95 % confidence intervals (CI95 %) were calculated, considering p<0,05 as statistically significant. Models were adjusted for potential confounders, including age and sex.

## RESULTS

A total of 116 children and adolescents were analyzed, with a predominance of females (62,1 %, n=72) compared to males (37,9 %, n=44), and a mean age of 6,98 ± 1,987 years. The prevalence of dental caries was 45,7 % in permanent teeth and 77,6 % in primary teeth. Regarding the consequences of the disease, 9,5 % of permanent teeth had been lost and 6,9 % of primary teeth had been extracted due to dental caries, while only 3,4 % of permanent and 2,6 % of primary teeth presented restorations. Additionally, pulp involvement was observed in 6,9 % of permanent teeth and 37,9 % of primary teeth. Finally, a single fistula was recorded in primary teeth, with no cases of ulcerations or abscesses in either dentition (Table 1).

**Table 1:** Dental Caries Experience (DMFT/dmft) and Clinical Consequences (PUFA/pufa) in Children from Rural Communities in the Peruvian Amazon.

Indicators	n	%
D	53	45,7
M	11	9,5
F	4	3,4
DMFT	1,56 ± 2.18*		
d	90	77,6
m	8	7,1
f	3	2,6
dmft	4,16 ± 4.0*	
P	8	6,9
U	0	0
F	0	0
A	0	0
p	44	37,9
u	0	0
f	1	0,9
a	0	0

Note: values expressed as mean ± standard deviation. DMFT/dmft = caries experience indices; PUFA/pufa = clinical consequences of untreated caries.

It was observed that underweight was the most frequent nutritional condition (80,2 %), while overweight and obesity accounted for smaller proportions. Regarding oral health, the prevalence of caries in primary teeth was high among underweight children (83,9 %) and reached 100 % in those with grade I and II obesity, whereas it was considerably lower in children with normal BMI (35,7 %). In permanent teeth, a higher frequency of caries was also observed in children with obesity, with all cases presenting lesions, compared to underweight (40,9 %) and overweight children (25 %) (Table 2).

**Table 2:** Body Mass Index-for-Age and Dental Caries in Children from Rural Communities in the Peruvian Amazon.

IMC/E	Total	Male	Female	Dental caries in primary teeth			Dental caries in permanent teeth	
				Yes	No	Yes	No
	n (%)	n (%)	n (%)	n (%)	n (%)	n (%)	n (%)
Underweight	93 (80,2)	57 (61,3)	36 (38,7)	78 (83,9)	15 (16,1)	38 (40,9)	55 (59,1)
Normal BMI-for-age	14 (12,1)	7 (50,0)	7 (50,0)	5 (35,7)	9 (64,3)	9(64,3)	5 (35,7)
Overweight	4 (3,4)	3 (75,0)	1 (25,0)	2 (50,0)	2 (50,0)	1 (25,0)	3 (75,0)
Obesity grade I	4 (3,4)	3 (75,0)	1 (25,0)	4 (100,0)	0 (0,0)	4 (100,0)	0 (0,0)
Obesity grade II	1 (0,9)	0 (0,0)	1 (100,0)	1 (100,0)	0 (0,0)	1 (100,0)	0 (0,0)
Total	116 (100,0)	72 (62,1)	44 (37,9)	90 (77,6)	26 (22,4)	53 (45,7)	63 (54,3)

Note: IMC/E = Body Mass Index-for-Age. Categories are defined according to WHO standards: Underweight = BMI-for-age < -2 SD; Normal BMI-for-age = -2 SD ≤ BMI-for-age ≤ +1 SD; Overweight = +1 SD < BMI-for-age ≤ +2 SD; Obesity grade I = +2 SD < BMI-for-age ≤ +3 SD; Obesity grade II = BMI-for-age > +3 SD.

The majority of children and adolescents evaluated had a moderate social risk (RSM) at 81,1 % (n=94), followed by low social risk (RSA) at 15,5 % (n=18) and high social risk (RMA) at 3,4 % (n=4). When disaggregated by sex, boys accounted for higher proportions in all categories, representing 62,8 % of RSM, 55,6 % of RSA, and 75,0 % of RMA, while girls represented 37,2 %, 44,4 %, and 25,0 %, respectively. Regarding dental caries, in primary teeth it was present in 75,0 % of children with RMA, 72,2 % with RSA, and 78,7 % with RSM, indicating a high frequency across all groups. For permanent teeth, caries occurred in 50,0 % of children with RMA, 44,4 % with RSA, and 45,7 % with RSM (Table 3).

**Table 3:** Waist Circumference-for-Age and Dental Caries in Children from Rural Communities in the Peruvian Amazon.

AC/A	Total	Male	Female	Dental caries in primary teeth			Dental caries in permanent teeth	
				Yes	No	Yes	No
	n (%)	n (%)	n (%)	n (%)	n (%)	n (%)	n (%)
RMA	4(3.4)	3(75.0)	1(25.0)	3(75.0)	1(25.0)	2(50.0)	2(50.0)
RSA	18(15.5)	10(55.6)	8(44.4)	13(72.2)	5(27.8)	8(44.4)	10(55.6)
RSM	94(81.1)	59(62.8)	35(37.2)	74(78.7)	20(21.3)	43(45.7)	51(54.3)
Total	116 (100,0)	72(62.1)	44(37.9)	90(77.6)	26(22.4)	53(45.7)	63(54.3)

Note: RMA = Very high risk of abdominal obesity; RSA = High risk of abdominal obesity; RSM = Low risk of abdominal obesity (reference group). AC/A = Waist Circumference-for-Age.

It was observed that most children had normal height-for-age (77.6%), while 19.0% had stunting and 3.4% had severe stunting. Regarding caries in primary teeth, prevalence was high among children with stunting (72.2%) and normal height-for-age (75.0%), and lower in those with severe stunting. In permanent dentition, children with stunting and severe stunting also showed higher caries frequency (44.4% and 45.7%, respectively), although all groups had a considerable proportion of affected individuals (Table 4).

**Table 4:** Height-for-Age and Dental Caries in Children from Rural Communities in the Peruvian Amazon.

H/A	Total	Male	Female	Dental caries in primary teeth			Dental caries in permanent teeth	
				Yes	No	Yes	No
	n (%)	n (%)	n (%)	n (%)	n (%)	n (%)	n (%)
Normal height-for-age	90(77.6)	3(75.0)	1(25.0)	3(75.0)	1(25.0)	2(50.0)	2(50.0)
Stunting	22(19.0)	10(55.6)	8(44.4)	13(72.2)	5(27.8)	8(44.4)	10(55.6)
Severe stunting	4(3.4)	59(62.8)	35(37.2)	74(78.7)	20(21.3)	43(45.7)	51(54.3)
Total	116(100.0)	72(62.1)	44(37.9)	90(77.6)	26(22.4)	53(45.7)	63(54.3)

Note: H/A = Height-for-Age. Categories are defined according to WHO Child Growth Standards: Normal height-for-age = Z-score between -2 SD and +2 SD; Stunting = Z-score between -3 SD and -2 SD; Severe stunting = Z-score < -3 SD.

The multivariate analysis showed that, for each additional year of age, the likelihood of presenting dental caries increased 2.5-fold in permanent dentition (OR=2,543; 95% CI: 1,780-3.633; p<0,001) and 1.7-fold in primary dentition (OR=1,746; 95% CI: 1,587-1,948; p=0,017). Regarding sex, girls had an 86% higher risk of caries in permanent teeth (OR=1,868; 95% CI: 0,340-2,217; p=0,914) and a 113% higher risk in primary teeth (OR=2,132; 95% CI: 0,848-5,292; p=0,108), although these differences were not statistically significant.

Concerning BMI, children with overweight/obesity showed a 3.8-fold higher risk of developing caries in permanent dentition (OR=3,875; 95% CI: 0,462-32,496; p=0,212) and, conversely, a 46% lower risk in primary dentition (OR=0,539; 95% CI: 0,087-3,325; p=0,506); however, neither association reached statistical significance. For abdominal circumference-for-age, children classified as high or very high risk for obesity had an OR of 0,968 (95% CI: 0,243-3,858; p=0,919) for permanent dentition and 0.864 (95% CI: 0,234-3,195; p=0,827) for primary dentition, indicating no statistically significant association between obesity risk by abdominal circumference and dental caries in either dentition. Finally, for height-for-age, children at risk of stunting had a 31% higher probability of caries in permanent teeth (OR=1,319; 95% CI: 0,101-1,009; p=0,520) and 76% higher in primary teeth (OR=1,757; 95% CI: 0,584-5,292; p=0,316), with no significant differences (Table 5).

**Table 5:** Association between Anthropometric Indicators and Dental Caries in Children from Rural Communities in the Peruvian Amazon.

Variable		Permanent teeth			Primary teeth		
		OR	95% CI	p	OR	95% CI	p
Age		2,543	1,780- 3,633	0,00	1,746	1,587 - 1,948	0,017
Sex	Male	Ref.	Ref.	Ref.	Ref.	Ref.	Ref.
	Female	1,868	0,340 - 2,217	0,914	2,132	0,848 - 5,292	0,108
BMI/A	Low weight (Underweight + Normal weight)	Ref.	Ref.	Ref.	Ref.	Ref.	Ref.
	High weight						
(Overweight + Obesity)	3,875	0,462 - 32,496	0,212	0,539	0,087 - 3,325	0,506	
CA/A	Low risk of obesity	Ref.	Ref.	Ref.	Ref.	Ref.	Ref.
	High risk of obesity + Very high-risk obesity	0,968	0,243 - 3,858	0.919	0,864	0,234-3,195	0,827
H/A	Weight appropriate for age	Ref.	Ref.	Ref.	Ref.	Ref.	Ref.
	At risk of stunting	1,319	0,101 - 1,009	0,52	1,757	0,584 - 5,292	0,316

-binary logistic regression; statistical significance p<0.05

## DISCUSSION

The results of our study show a prevalence of dental caries of 77.6% in primary dentition and 46.6% in permanent dentition, consistent with figures reported in other South American indigenous communities. In Brazil, a systematic review reported prevalence’s ranging from 50% in children aged 18-36 months to 100% in older adults ^15^. In Ecuador, only 7.6% of indigenous Amazonian schoolchildren had caries-free primary teeth (mean dmft: 6.40), and 6% were free of caries in permanent dentition (mean DMFT: 4.47) ^16^. Among Amazonian indigenous communities in Venezuela, the mean DMFT index was 2.97 (SD ±3.7), classified as moderate, reflecting a similar pattern of high caries frequency in populations with comparable sociocultural conditions and access to oral healthcare ^17^.

Pulpal involvement was observed in 6.9% of permanent teeth and 37.9% of primary teeth in children aged 5 to 11 years, exceeding reported values for primary teeth in other Latin American contexts (20-30%) ^18^^-^^21^. This may be related not only to biological factors but also to conditions specific to rural Amazonian areas, such as limited availability of specialized dental services, low frequency of preventive check-ups, frequent consumption of fermentable carbohydrates, and geographical and cultural barriers to timely treatment ^18^^,^^19^. In contrast, pulpal involvement in permanent teeth (6.9%) was similar to that reported in schoolchildren of comparable age ^20^^,^^21^. Its presence at such early ages is concerning as it may compromise future oral health and negatively affect nutrition, well-being, and academic performance.

## BMI AND DENTAL CARIES

In this study, underweight was the most frequent nutritional condition (80.2%), and the prevalence of caries was high both in underweight children (83.9%) and in those with obesity (100%), compared to children with adequate BMI (35.7%). These findings are consistent with reports from Amazonian indigenous communities, where caries and malnutrition coexist critically ^22^^,^^23^. Children with overweight or obesity had a 3.8-fold higher risk of caries in permanent teeth and a 46% lower risk in primary teeth, although these associations were not statistically significant. These results align with previous evidence reporting positive associations between obesity and caries in permanent dentition, mainly attributed to cariogenic diets rich in fermentable sugars and shared unhealthy dietary habits ^24^. Recent reviews have identified a positive correlation between elevated BMI and greater caries severity in schoolchildren, though methodological heterogeneity and cross-sectional designs limit definitive conclusions ^25^. However, evidence remains contradictory. A meta-analysis in a Brazilian population including children and adolescents found no consistent association between overweight/obesity and dental caries. Interestingly, analysis of permanent dentition using CDC 2000 growth curves showed that eutrophic children had higher caries experience compared to obese children (OR=2.53; 95% CI: 1.49-4.29; p=0.0006) ^26^. Furthermore, an international systematic review concluded that evidence is inconsistent, with positive, negative, or neutral results depending on the study. Among studies with lower risk of methodological bias, most found no significant association, while a few reported a positive relationship between BMI and caries in older children ^27^.

## ABDOMINAL CIRCUMFERENCE FOR AGE AND DENTAL CARIES

Analysis of abdominal circumference for age in children with high or very high obesity risk did not show a statistically significant association with caries in either permanent or primary dentition. These results are consistent with previous studies that did not find a direct relationship between central obesity and dental caries in children. For instance, a study in English children aged 4-6 years found no association between obesity and caries, regardless of whether BMI or abdominal circumference was used as the obesity indicator ^28^. A possible explanation for the lack of association in our study is that central obesity, as measured by abdominal circumference, does not necessarily reflect dietary behaviors specifically associated with dental caries. While obesity and caries share risk factors such as sugar intake, their relationship may be mediated by other factors, including oral hygiene, access to dental services, and socioeconomic status. Indeed, a study in adolescents from the United Arab Emirates found that although obesity measured by abdominal circumference was significantly associated with dental caries, this relationship was mediated by oral health habits and socioeconomic factors ^29^.

## HEIGHT-FOR-AGE AND DENTAL CARIES

Children at risk of stunting showed higher odds of caries in both permanent and primary dentition, although differences were not statistically significant. These findings are consistent with previous studies reporting weak or null associations between stunting and dental caries. For example, a study in Japan with 3,576 primary schoolchildren found that children with height-for-age below -3.00 SD had higher mean permanent dental caries (MR: 2.18; 95% CI: 1.03-4.64), but the association was not statistically significant after adjusting for confounders ^30^. A possible explanation is that height-for-age primarily reflects chronic nutrition and linear growth, whereas dental caries is more influenced by immediate factors such as diet, oral hygiene, and fluoride exposure. Thus, stunting may indicate chronic undernutrition but does not necessarily translate into increased caries risk, especially when oral hygiene practices are adequate ^31^.

## LIMITATIONS

This study has some limitations. Due to its cross-sectional design, causal relationships between anthropometric indicators and dental caries cannot be established. Additionally, the sample included only children under 11 years from rural communities in Satipo, limiting generalizability to other populations, as factors such as diet, access to health services, and oral hygiene education may have influenced the results. Measurement biases are also possible, as dental caries evaluation was conducted in the field and anthropometric indicators were obtained at a single time point without long-term follow-up.
